# Hierarchical N-Doped Porous Carbons for Zn–Air Batteries and Supercapacitors

**DOI:** 10.1007/s40820-019-0364-z

**Published:** 2020-01-10

**Authors:** Beibei Guo, Ruguang Ma, Zichuang Li, Shaokui Guo, Jun Luo, Minghui Yang, Qian Liu, Tiju Thomas, Jiacheng Wang

**Affiliations:** 1 State Key Laboratory of High Performance Ceramics and Superfine Microstructure, Shanghai Institute of Ceramics, Chinese Academy of Sciences, Shanghai, 200050 People’s Republic of China; 2 Center of Materials Science and Optoelectronics Engineering, University of Chinese Academy of Sciences, Beijing, 100049 People’s Republic of China; 3 School of Materials Science and Engineering, Shanghai University, Shanghai, 200444 People’s Republic of China; 4 Solid State Functional Materials Research Laboratory, Ningbo Institute of Materials Technology and Engineering, Chinese Academy of Sciences, Ningbo, 315201 People’s Republic of China; 5 Department of Metallurgical and Materials Engineering, Indian Institute of Technology Madras, Adyar, Chennai, Tamil Nadu 600036 India

**Keywords:** Porous carbon, Ball milling, Nitrogen doping, Oxygen reduction reaction, Zn–air battery, Supercapacitor

## Abstract

Nitrogen-doped carbon materials with a large specific surface area, high conductivity, and adjustable microstructures have many prospects for energy-related applications. This is especially true for N-doped nanocarbons used in the electrocatalytic oxygen reduction reaction (ORR) and supercapacitors. Here, we report a low-cost, environmentally friendly, large-scale mechanochemical method of preparing N-doped porous carbons (NPCs) with hierarchical micro-mesopores and a large surface area via ball-milling polymerization followed by pyrolysis. The optimized NPC prepared at 1000 °C (NPC-1000) offers excellent ORR activity with an onset potential (*E*_onset_) and half-wave potential (*E*_1/2_) of 0.9 and 0.82 V, respectively (vs. a reversible hydrogen electrode), which are only approximately 30 mV lower than that of Pt/C. The rechargeable Zn–air battery assembled using NPC-1000 and the NiFe-layered double hydroxide as bifunctional ORR and oxygen evolution reaction electrodes offered superior cycling stability and comparable discharge performance to RuO_2_ and Pt/C. Moreover, the supercapacitor electrode equipped with NPC prepared at 800 °C exhibited a high specific capacity (431 F g^−1^ at 10 mV s^−1^), outstanding rate, performance, and excellent cycling stability in an aqueous 6-M KOH solution. This work demonstrates the potential of the mechanochemical preparation method of porous carbons, which are important for energy conversion and storage.

**Electronic supplementary material:**

The online version of this article (10.1007/s40820-019-0364-z) contains supplementary material, which is available to authorized users.

## Highlights

- Hierarchical N-doped porous carbons (NPCs) with large surface area and controllable N-doping are synthesized by ball milling, followed by pyrolysis.
- As a Zn–air battery cathode, NPCs have comparable discharge performance to precious metal catalysts and more stability.
- NPCs also exhibit an excellent specific capacity and cycling stability when used as supercapacitor electrodes.

**Electronic supplementary material:**

The online version of this article (10.1007/s40820-019-0364-z) contains supplementary material, which is available to authorized users.

## Graphical abstract

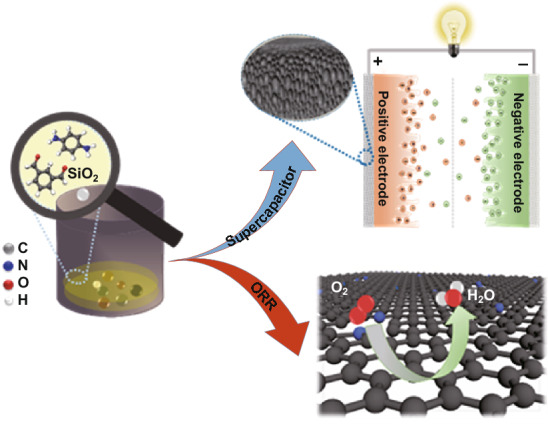

## Introduction

Traditional fossil resources are progressively being replaced by sustainable and clean energy sources. However, the movement toward a greener economy requires energy storage. Electrochemical energy devices, such as fuel cells, metal–air batteries, and supercapacitors, are expected to play a crucial role in the transition to a sustainable future [1]. The oxygen reduction reaction (ORR) is an important cathodic reaction in fuel cells and metal–air batteries. The development of highly efficient and stable ORR catalysts could resolve the bottleneck of fuel cells and metal–air batteries [2, 3]. Noble metal Pt and Pt-based alloys are the most efficient ORR electrode materials. However, although Pt-based catalysts have high ORR activity, there are several associated hindrances, such as CO poisoning, metal dissolution, poor stability, scanty supply of noble metals, and high costs [4]. To resolve these issues, extensive efforts have been devoted to designing high-activity and more stable non-precious metal-based ORR catalysts [5, 6]. In 2009, Gong prepared a metal-free catalyst (nitrogen-doped VA-CNT) as an ORR electrocatalyst, and its performance exceeded that of commercial Pt/C [7]. Subsequently, carbon-based electrocatalysts with other heteroatoms (such as B, P, and S) have also been developed, which exhibited excellent ORR catalytic activity [8–10]. An excellent ORR electrocatalyst typically exhibits the following characteristics: (1) a large number of active sites, (2) high specific surface area, which increases the three-phase boundary and active surface area [11], (3) outstanding conductivity, which accelerates electron transfer during electrocatalysis, and (4) excellent stability, which must be considered in practical applications. The high specific surface area and high conductivity considered desirable here are also required in supercapacitors [12].

Among the many metal-free catalysts, N-doped porous carbon (NPC) could be used in precious metal catalysts due to its high catalytic activity and specific surface area, good electrical and thermal conductivity, and easily adjustable microstructure [2, 9, 13, 14]. Furthermore, NPCs could improve the capacitance of the catalyst via surface Faradaic reactions [15]. Micropores play an important role in the adsorption of ions [16, 17], while mesopores accelerate the transport of ions to the bulk of the material [18, 19]. Hierarchical micro-mesoporous structures can provide numerous accessible active sites and accelerate the mass transport of ORR-related species (O_2_, protons, and H_2_O) [20–22]. Li et al. demonstrated that N-doped hierarchical porous carbon (N-HPC) has a larger specific surface area, faster charge transferability, and better surface wettability than undoped HPC [23, 24].

The pyrolysis of N-containing organic polymers is a very simple and efficient method of preparing NPCs and allows a certain degree of morphological control [25–27]. The porosity and catalytic activity of the NPC can be optimized by adjusting the carbonization temperature [27]. However, the synthesis of N-containing polymers often requires the use of catalysts or large amounts of organic solvents to promote the reaction. Thus, the synthesis process is relatively complicated [25–27]. In addition, the mass use of organic solvents may also cause harm to both humans and the environment; therefore, the process requires further improvement. Compared to the various strategies for preparing NPCs, ball milling induces chemical polymerization reactions by mechanical force, which is a scalable, solvent-free, low-cost, environmentally and economically sustainable approach [28–30]. Additionally, ball mills are common in modern industrial production; therefore, preparing NPCs by a mechanochemical method is more likely to result in a technology that can be translated.

Herein, we explore a mechanochemical strategy of synthesizing N-containing polymer precursors using planetary ball mills without solvents and catalysts with isophthalaldehyde and p-phenylenediamine as the carbon sources. During ball milling, the amine and aldehyde groups condense, leading to the formation of polyamine-based polymers [31]. The pyrolysis of such polymer precursors would produce NPCs with large numbers of micropores [27, 31]. To optimize the pore structure, we introduced nanosized SiO_2_ spheres as a template to produce mesoporous structures during ball milling. The hierarchical NPC is obtained by the pyrolysis of the polymer precursor containing SiO_2_, followed by the removal of the SiO_2_ template. The resultant NPC has a large specific surface area (641–1013 m^2^ g^−1^), the abundant micropores and mesopores which are beneficial for the adsorption and transport of ions and reactants. NPC-1000, which was prepared at 1000 °C, exhibits excellent ORR activity in KOH solutions. The onset potential (*E*_onset_, defined as the potential when the current density reaches 0.1 mA cm^−2^) and half-wave potential (*E*_1/2_) of NPC-1000 are only 30 mV lower than those of commercial Pt/C. However, the stability and methanol tolerance of NPC-1000 are much better than that of Pt/C. Zn–air batteries (ZABs) with an NPC-1000 cathode achieve a high open-circuit voltage of 1.43 V, and comparable discharge performance and energy density to those of Pt/C. Its cycling stability is also better than that of Pt/C. Furthermore, owing to the higher nitrogen content and hierarchical pore structure, NPC-800 exhibits outstanding capacitive behavior as a supercapacitor electrode (256 F g^−1^ at 0.5 A g^−1^ and 431 F g^−1^ at 10 mV s^−1^) and excellent cycling stability (98.7% retention after 10,000 cycles at 10 A g^−1^) in an aqueous 6-M KOH electrolyte.

## Experimental

### Preparation of Nitrogen-Doped Porous Carbon

All chemical reagents are of analytical-grade and used without further purification. To prepare the NPC, isophthalaldehyde (1.13 g, 8.5 mmol), p-phenylenediamine (0.91 g, 8.5 mmol), and 2.04 g of 12-nm silica spheres (mass ratio of SiO_2_ to the carbon sources is 1:1) are placed in a zirconium oxide grinding jar with twenty-four 5-mm zirconium oxide grinding balls. The mixture is milled at 500 rpm for 5 h using an XGB2 planetary ball mill to form a yellow precursor. The precursor is subsequently dried at 80 °C overnight and pyrolyzed in a tube furnace at 700–1100 °C for 2 h at a heating rate of 5 °C min^−1^ under an argon atmosphere (denoted as NPC-*T*, where *T* is the pyrolysis temperature). The silica template is removed by leaching with 2 M NaOH at 80 °C for 4 h. The above leaching process is repeated twice to ensure that the silica is completely removed. The final product is washed with water and ethanol until the pH of the filtrate is approximately 7 and then dried at 80 °C.

NPC-1000-0 and NPC-1000-2 are synthesized following the approach described above, where 1000 indicates that the pyrolysis temperature is 1000 °C, and 0 and 2 indicate the mass of SiO_2_, which is zero and two times that of the total carbon sources (isophthalaldehyde and p-phenylenediamine).

Fe-doped NPC (Fe-NPC) is also synthesized following the same procedure as NPC-1000, with the addition of 0.081 g of FeCl_3_ during ball milling.

### Material Characterization

Powder X-ray diffraction (XRD) analysis is conducted using a D8 ADVANCE instrument with Cu K_α_ radiation (40 kV, 60 mA). The morphologies are characterized from scanning electron microscopy (SEM) images obtained using a field emission scanning electron micro-analyzer (FEI Magellan 400), and transmission electron microscopy (TEM; JEM-2100F). Raman spectra are obtained with a DXR Raman Microscope (Thermal Scientific Co., USA) with an excitation length of 532 nm. Two spectra are obtained for each sample to ensure accuracy. Nitrogen adsorption–desorption isotherms are measured at − 196  °C using an ASAP 2010 accelerated surface area and pore size analyzer system (Micrometitics, Norcross, GA). The specific surface areas are obtained following the multipoint Brunauer–Emmett–Teller (BET) method. The pore-size distribution curves, pore volume, and pore diameter are calculated using the adsorption branch of the isotherms following the Barrett–Joyner–Halenda (BJH) method. X-ray photoelectron spectroscopy (XPS) measurements are used to analyze the surface of the samples with an ESCALAB 250 X-ray photoelectron spectrometer and Al K_α_ (*hν* = 1486.6 eV) radiation.

### Electrochemical Measurements

The catalyst ink is prepared by blending the catalyst powder (5 mg) with 20 μL of a Nafion solution (5 wt%), 500 μL of ethanol, and 500 μL of deionized water in an ultrasonic bath, and 20 μL of the catalyst ink is pipetted and spread onto the glassy carbon (GC) electrode (catalyst loading amount of 0.5 mg cm^−2^). For comparison, commercial 20 wt% platinum on Vulcan carbon black (Pt/C) with the same loading amount is analyzed under the same conditions.

All electrochemical measurements are conducted in a conventional three-electrode cell using a CHI760E electrochemical workstation (CH Instrument) at room temperature. A GC electrode (5.0 mm diameter) is used as the working electrode, and a saturated calomel electrode (SCE) and graphite rod are used as the reference and counter electrodes, respectively. The ORR electrochemical experiments are conducted in an O_2_-saturated 0.1 M KOH electrolyte. To remove the capacitive current of the working electrode, the background current is measured by running the above electrodes in N_2_-saturated 0.1 M KOH. In the reported ORR polarization curves, the background current is subtracted from the capacitive current. All measured potentials are converted to the potentials versus the reversible hydrogen electrode (RHE) based on *E*_RHE_ = *E*_SCE_ + 0.2415 + 0.059 × pH.

The rotating ring-disk electrode (RRDE) tests are conducted on a GC disk (0.2475 cm^2^) surrounded by a Pt disk (0.1866 cm^2^). The ring potential is maintained at 1.21 V (vs. RHE) to detect the production of H_2_O_2_ species. The number of electrons transferred (*n*) and percentage of H_2_O_2_ released (%H_2_O_2_) during the ORR process are calculated using Eqs. 1 and 2, respectively:

1 $$n = 4\frac{{I_{\text{d}} }}{{I_{\text{d}} + I_{\text{r}} /N}}$$

2 $$\%{\text{H}}_{2} {\text{O}}_{2} = 200\frac{{I_{\text{r}} /N}}{{I_{\text{d}} + I_{\text{r}} /N}}$$

where *I*_d_ is the disk current, *I*_r_ is the ring current, and *N* (the value is 0.37) is the collection efficiency of the Pt ring electrode.

### Zn–Air Battery Measurements

Liquid Zn–air battery (ZAB) tests are conducted using a homemade Zn–air cell. The air cathode consists of a hydrophobic carbon paper with a gas diffusion layer on the air-facing side and a catalyst layer on the water-facing side. The loading amount for all catalysts is 0.25 mg cm^−2^. A polished Zn plate with a thickness of 0.3 mm is used as the anode. The electrolyte used for ZAB is 6.0 M KOH containing 0.20 M Zn(Ac)_2_. To evaluate the potential of NPC-1000 in a real device, NPC-1000 and NiFe-LDH (NiFe-layered double hydroxide) with a mass ratio of 1:1 are used as the air cathode. NiFe-LDH achieves excellent OER activity, which reduces the charging voltage of the Zn–air battery. Cycling tests are conducted using a Land CT2001A system, and each discharge and charge period are set to be 30 min. The charge–discharge polarization curve is tested using a PINE electrochemical workstation (Pine Research Instrumentation, USA). For comparison, a mixture of noble metal Pt/C and RuO_2_ (mass ratio 1:1) with the same loading amount is also tested.

### Supercapacitor Measurements

Supercapacitor measurements are also conducted in a conventional three-electrode cell using a CHI760E electrochemical workstation (CH Instrument) at room temperature. The ink and electrode are prepared following the same method as those for ORR testing. The electrolyte used for the supercapacitor measurement is 6-M KOH. The gravimetric-specific capacitance (*C*_m_ in F g^−1^) based on the galvanostatic charge/discharge profile and CV curve is calculated using Eq. 3:

3 $$C_{\text{m}} = \frac{i \times \Delta t}{m \times \Delta V}$$

where *i* (*A*) is the discharge current, ∆*V* (*V*) is the potential window within the discharge time ∆*t* (s), and *m* (g) is the mass loading of the electrode material.

4 $$C_{m} = \frac{1}{{m\nu \left| {V_{\text{a}} - V_{\text{b}} } \right|}}\mathop \smallint \limits_{{V_{\text{b}} }}^{{V_{\text{a}} }} i\left( V \right){\text{d}}V$$

where *m* is the mass loading of the electrode material, *ν* is the scan rate, *V*_a_ and *V*_b_ are the cathodic and anodic potentials, respectively, and *i* (*V*) is the current response at potential *V*.

## Results and Discussion

**Fig. 1:**
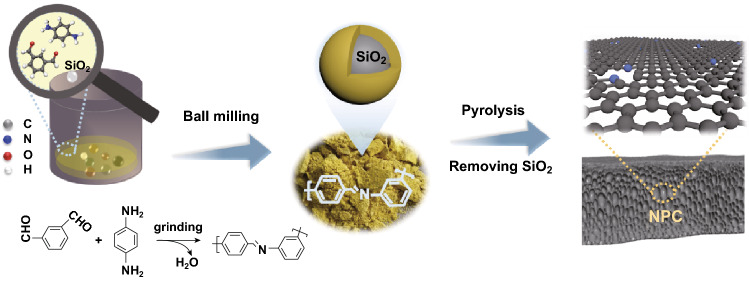
Schematic representation of the preparation of N-doped porous carbon (NPC) via a mechanochemical route

**Fig. 2:**
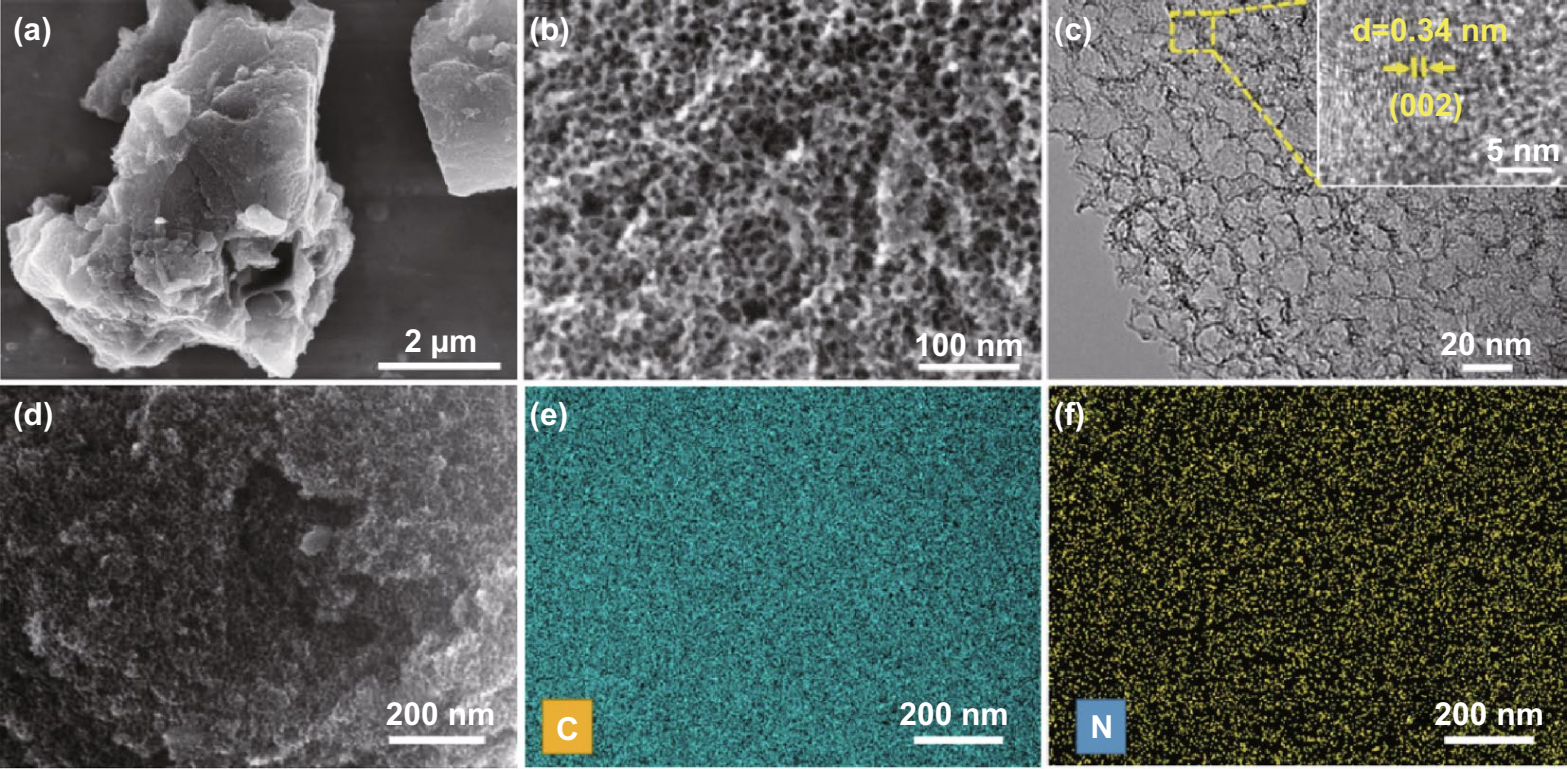
**a**, **b** SEM, and **c** TEM images of NPC-1000, **d**–**f** SEM image of NPC-1000 and corresponding elemental mapping images of C and N

Figure 1 shows the nitrogen-doped porous carbon preparation process. First, the condensation reaction of isophthalaldehyde and p-phenylenediamine produces a yellow polymer precursor, and the SiO_2_ nanospheres are uniformly dispersed during ball milling. Second, high-temperature pyrolysis combined with the removal of the SiO_2_ template leads to the formation of the sheetlike NPCs with large numbers of micropores and mesopores (Fig. 2a, b). The porous structure is further verified by the TEM images (Fig. 2c). The high-resolution TEM images show lattice fringes with lattice spacing of 0.34 nm consistent with the (002) facet of graphitic carbon. The distribution of nitrogen can be confirmed by the elemental mapping (Fig. 2e, f) and indicates that nitrogen atoms are successfully doped into the carbon skeleton, which is essential for good ORR activity and wettability [32].

**Fig. 3:**
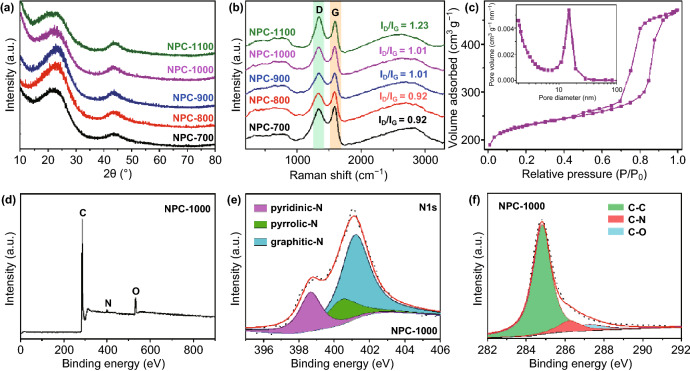
**a** XRD patterns and **b** Raman spectra of different NPCs, **c** nitrogen adsorption–desorption isotherms of NPC-1000 (inset: pore-size distribution), **d** XPS spectra, and **e**, **f** high-resolution N 1s and C1s spectra of NPC-1000

The XRD patterns are studied to investigate the microstructures and textural properties of NPCs. Figure 3a shows the XRD patterns of the NPCs carbonized at different temperatures. The two broad diffraction peaks at approximately 2*θ *= 23° and 44° are indexed to the (002) and (101) diffraction of graphitic carbon, respectively. The high intensity in the low-angle region of the XRD patterns is attributed to the high number of pores in the NPC samples [33]. Raman spectroscopy is a nondestructive, fast, high-resolution testing method that provides the most structural and electronic information regarding carbon materials [34]. As shown in Fig. 3b, the two peaks at approximately 1340 and 1590 cm^−1^, corresponding to the D and G peaks, are characteristic of the defective graphitic and graphitic layers, respectively. The significant G peak indicates the presence of a graphitized carbon structure, consistent with TEM and XRD results. The ratio of the intensity of the D/G bands (*I*_D_/*I*_G_) can be used to characterize the defects in the carbon material. The *I*_D_/*I*_G_ value gradually increases as the carbonization temperature increases, indicating a higher number of defects at higher temperatures. The porosity of the as-prepared material is obtained from the N_2_ adsorption–desorption isotherm curves. Figure 3c shows the combined type-I/IV isotherms that indicate the presence of micropores (< 2 nm) and mesopores (2–50 nm). The micropores are mainly derived from the carbonization of the polymer, and the mesopores are produced by the removal of silica nanospheres. As indicated by the pore-size distribution curves in the inset of Fig. 3c, uniform mesopores are present, centering at approximately 15 nm. To explore the effect of temperature and silica template content on the porous structure, the N_2_ adsorption–desorption isotherms and pore-size distribution curves of NPC-800, NPC-1100, NPC-1000-0 (without SiO_2_), and NPC-1000-2 (mass ratio of SiO_2_ to carbon source is 2) are also obtained (Fig. S1), and the results are presented in Table S1. The results show that the Brunauer–Emmett–Teller surface area (*S*_BET_) and micropore area gradually improve with increasing temperature. That is, high-temperature carbonization will lead to the formation of more micropores. The generation of numerous micropores and mesopores will create more edge sites and defects, which is consistent with the change of *I*_D_/*I*_G_ values in the Raman spectra. The hierarchical micro-mesopore structure facilitates electron and ion transport during the reaction [21, 35, 36]. The micropore area (118 m^2^ g^−1^) and specific surface area (140 m^2^ g^−1^) of NPC-1000-0 are almost the same, confirming that the mesopores in NPC-1000 are derived from the removal of the SiO_2_ template and the micropores are derived from the pyrolysis of polymers. The use of a higher SiO_2_ content for NPC-1000-2 results in a smaller specific surface area due to the collapse of the pore structure.

The surface chemical compositions and surroundings of the as-prepared samples are investigated by XPS analysis, and an XPS survey scan of the NPC-1000 is presented in Fig. 3d, which shows the three peaks attributable to C, N, and O. This suggests that N atoms are still present in the carbon subjected to high-temperature pyrolysis. The high-resolution N 1*s* spectra show three peaks for pyridinic N (~ 398.7 eV), pyrrolic N (~ 400.5 eV), and graphitic N (~ 401.2 eV) [21, 37]. Previous studies demonstrated that pyridinic N and graphitic N are beneficial for improving the electrochemical performance, as N-doping could modify the electronic structure and improve the surface hydrophilicity, conductivity, and adsorption performance, as well as the Fermi levels of the adjacent carbon atoms [38–41]. The presence of C–N in the high-resolution C 1*s* spectra further confirms that N atoms are doped into the carbon skeleton. For comparison, the XPS of NPC-800 and NPC-1100 are also measured (Fig. S2, Table S2), and the results show that the proportions of pyridinic N, pyrrolic N, and graphitic N are almost the same after pyrolysis at different temperatures. NPC-800, NPC-1000, and NPC-1100 have nitrogen contents of 4.3, 2.2, and 1.1 at.%, respectively. That is, the higher the pyrolysis temperature, the lower the nitrogen content. Additionally, the oxygen-containing functional groups on the carbon surface can also affect the doping of N and promote the introduction of N to the highly active sites of the carbon lattice [42].

**Fig. 4:**
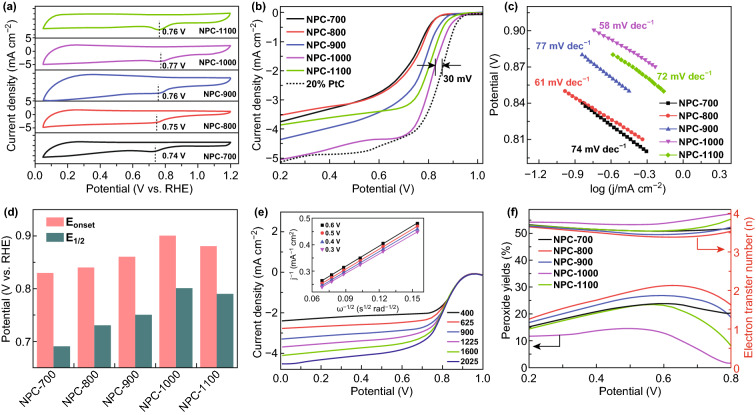
**a** Cyclic voltammograms (CVs), **b** ORR polarization curves (sweep rate: 10 mV s^−1^; rotation speed: 1600 rpm) of the as-prepared catalysts in an O_2_-saturated 0.1-M KOH electrolyte, **c** Tafel plots derived from **b**, **d** comparison of the onset (*E*_onset_) and half-wave (*E*_1/2_) potentials of different catalysts, **e** LSV curves of NPC-1000 at different rotation speeds (insert shows the corresponding Koutecky–Levich plots), **f** peroxide yield and electron transfer number of the as-prepared catalysts at various potentials based on the RRDE data

The ORR activity of all NPCs is first evaluated in 0.1 M KOH at room temperature following the rotating disk electrode (RDE) method. For comparison, commercial Pt/C is also tested. As shown in Fig. 4a, there is a significant reduction peak in the CV curves of all samples collected in the O_2_-saturated 0.1 M KOH solution. The ORR peak potential of NPC-1000 is the most positive, suggesting that its activity is optimum. The linear sweep voltammogram (LSV) curves further validate the superior electro-activity of NPC-1000 with higher onset (*E*_onset_ = 0.9 V) and half-wave (*E*_1/2_ = 0.82 V) potentials; these values are only 30 mV lower than those of commercial Pt/C. The higher limiting current density of NPC-1000 than that of the other catalysts indicates a faster diffusion rate. The excellent ORR activity of NPC-1000 is also indicated by the smaller Tafel slope of 58 mV dec^−1^. Figure 4d compares the onset and half-wave potentials of the catalysts synthesized at different temperatures and indicates that the ORR activity first increases and then decreases with an increase in the pyrolysis temperature. The RDE tests of NPC-1000 are conducted at various rotating rates, and the results fitted by the Koutecky–Levich (K–L) plot are the inverse of the current density (*j*^−1^) as a function of the inverse of the square root of the rotating speed (*ω*^−1/2^) at different potentials (Fig. 4e). The calculated electron transfer number is approximately 3.7, based on the K–L equation, suggesting that the NPC-1000-catalyzed ORR process is a primary four-electron reaction, rather than a two-electron reaction. Rotating ring-disk electrode (RRDE) tests are also conducted to calculate the electron transfer number (*n*) and detect the generation of the two-electron product, H_2_O_2_ (Fig. 4f). The lower peroxide yield and *n* of approximately 4 further confirm that NPC-1000 performs better in the ORR. A long-term test is necessary to evaluate the durability of the catalysts. The chronoamperometric responses of NPC-1000 and Pt/C catalysts are measured at 0.4 V (vs. RHE) and 1600 rpm and show that NPC-1000 is more durable than Pt/C over a long period (Fig. S3a). Moreover, NPC-1000 is more tolerant to methanol (Fig. S3b).

The effect of specific surface area and pore structure on the catalytic activity of ORR is also investigated by changing the amount of SiO_2_ template particles. As shown in Fig. S4, NPC-1000 performs better than NPC-1000-0; therefore, a high specific surface area and the presence of mesopores in the hierarchical pore structure are essential for improving ORR. The higher current density of NPC-1000 than that of NPC-1000-2 indicates that the pore structure impacts the diffusion process. The collapse of the NPC-1000-2 mesoporous structure hinders diffusion, resulting in a lower limiting current density than that of NPC-1000.

Our results are consistent with results that demonstrated that nitrogen doping (especially pyridinic and graphitic nitrogen) plays an important role in ORR electrocatalysis [38–40]. Furthermore, for NPCs, a large specific surface area is beneficial for more active sites and offering good electrical conductivity, which can accelerate electron transport during the reaction process. Considering the above, we speculate that the excellent ORR activity of NPC-1000 is mainly attributed to the high density of active sites, good conductivity, and hierarchical pore structure. First, although the nitrogen contents of NPC-700, NPC-800, and NPC-900 are higher than that of NPC-1000, their low specific surface areas resulted in the incomplete exposure of active sites. Second, although NPC-1100 has a higher specific surface area and conductivity, its nitrogen content is insufficient, resulting in an insufficient number of active sites. Finally, the hierarchically micro-mesoporous structure accelerates the transport of electrons and ions. Therefore, NPC-1000 offers the best ORR activity among all NPCs.

This mechanochemical method is also suitable for preparing porous carbons with metal doping. For example, when Fe ions are incorporated during ball milling followed by pyrolysis, the resulting Fe-NPC catalyst exhibits outstanding ORR activity that is comparable to that of commercial Pt/C (Fig. S5). This high ORR activity may be due to the formation of Fe–N_*x*_ sites, which is highly efficient for ORR in an alkaline solution [43–45].

**Fig. 5:**
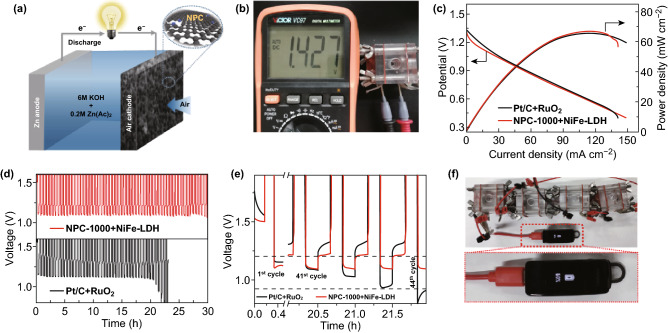
**a** Schematic illustration of the Zn–air battery with NPC-1000 as the air cathode, **b** image of the ZAB with a measured open-circuit voltage of 1.43 V, **c** discharge polarization curves and corresponding power-density plots, **d**, **e** charge/discharge cycling at 10 mA cm^−2^ (0.5 h for each cycle) for a rechargeable ZAB with NPC-1000 and NiFe-LDH as the air cathode, **f** image of a Bluetooth headset powered by four ZABs in series

To further investigate the performance of NPC in a real device, we conducted a test on ZABs using NPC-1000 and NiFe-LDH as the air cathode and Zn foil as the anode. KOH (6.0 M)-containing Zn(Ac)_2_ (0.20 M) is used as an electrolyte (Fig. 5a). For comparison, a mixture of Pt/C and RuO_2_ (mass ratio 1:1) with the same loading amount is also tested under the same conditions. As shown in Fig. 5b, the open-circuit potential of the ZAB with NPC-1000 is approximately 1.43 V. The discharge curves and corresponding power density of the ZAB catalyzed by NPC-1000 are comparable to those of Pt/C (Fig. 5c), suggesting the superior activity of NPC-1000. Galvanostatic discharge and charge measurements are taken to evaluate the cycling stability (Fig. 5d). The discharge voltage of Pt/C and RuO_2_ decreases greatly after 20 h, while that of the NPC-1000-equipped battery remained stable. This result is clearer in Fig. 5e, where the discharge voltage of NPC-1000 is lower than that of the Pt/C and RuO_2_ electrode in the first cycle, and almost the same in the 41st cycle. However, after 44 cycles, the discharge voltage of the Pt/C and RuO_2_-equipped battery decreases rapidly, but the voltage drop of NPC-1000 is negligible. A Bluetooth headset could be charged using four ZABs in series with NPC-1000 (Fig. 5f), demonstrating its promising application in rechargeable ZABs.

**Fig. 6:**
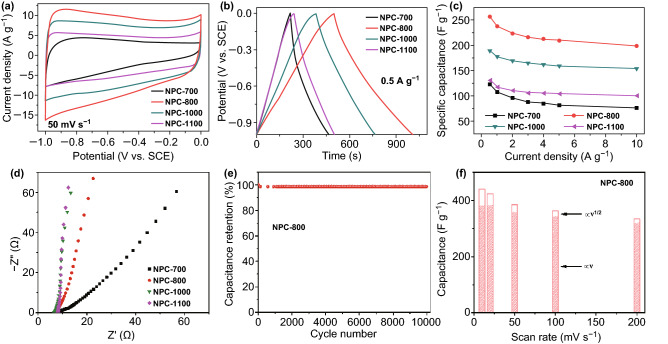
**a** CV curves at 50 mV s^−1^, **b** GCD curves at 0.5 A g^−1^, **c** variations in the specific capacitances at different current densities and **d** Nyquist plots of NPC-700, NPC-800, NPC-1000, and NPC-1100 in a 6-M KOH electrolyte, **e** cycling performance of NPC-800 at a current density of 10 A g^−1^, **f** deconvolution of the charge contribution as a function of the scan rate

As well as ORR electrocatalysis, the as-prepared NPC with high specific surface area, hierarchical micro-mesoporous structure, and nitrogen doping could also be used in supercapacitors. To demonstrate the supercapacitor performances, we conducted cyclic voltammetry (CV), galvanostatic charge–discharge (GCD), electrochemical impedance spectroscopy (EIS), and long-term cycling stability analysis in a three-electrode configuration. First, the CV curves of NPC-800, NPC-1000, and NPC-1100 exhibit a rectangular-like shape without any pronounced redox peaks (Fig. 6a), indicating the highly electric double-layer capacitive (EDLC) nature and small internal, interfacial, and contact resistances [46]. Unlike that of NPC-700, the CV curves of NPC-800, NPC-1000, and NPC-1100 are all almost rectangular, even at a scan rate of 200 mV s^−1^ (Fig. S6), and the GCD curves are all quasi-triangular (Figs. 6a and S7), indicating a higher discharge rate [46, 47].

The specific capacitance of the NPC-800 electrode calculated according to the corresponding CV curves is 431 F g^−1^ at 10 mV s^−1^, which is higher than that of the other electrodes. This suggests better capacitive performance when NPC-800 is used in supercapacitors. This capacitance is also higher than those for carbon-based materials reported in the literature [48–50]. All GCD curves exhibit a similar, almost symmetrical, triangular shape, indicating outstanding capacitor-like features, reversibility, and rapid charge transfer. Under the same conditions, the longer charging–discharging duration indicates a higher specific capacity. That is, NPC-800 has a higher specific capacity (256 F g ^−1^ at 0.5 A g ^−1^). The capacities of NPC-700, NPC-800, NPC-1000, and NPC-1100 are 61%, 78%, 81%, and 76%, respectively, following a 20-fold increase in the current density from 0.5 to 10 A g^−1^ (Fig. 6c). The higher capacity retention rates of NPC-800 and NPC-1000 under different current densities indicate their excellent rate performances. Excluding NPC-700, all Nyquist plots are measured by EIS (Fig. 6d) and present almost vertical lines in the low-frequency region, indicating nearly ideal capacitive behavior, and rapid ion diffusion and transport [51]. Additionally, no distinct semicircles are observed in the high-frequency region, demonstrating a low charge-transfer resistance [52]. After 10,000 cycles of constant current charge–discharge at a current density of 10 A g^−1^, 98.7% of the initial specific capacity of NPC-800 is maintained (Fig. 6e). This confirms the excellent cycling stability of this material. To better understand the charge storage procedures, the dependence of the current (*i*) on the scan rate (*ν*) is analyzed. The current at a specific potential (*i* (*V*)) is a combination of capacitive (*k*_1_*ν*) and diffusion-limited (*k*_2_*ν*^1/2^) processes, according to Eq. 5 [53, 54]:

5 $$i\left( V \right) \, = \, k_{1} \nu \, + \, k_{2} \nu^{1/2}$$

where *k*_1_ and *k*_2_ are fitting parameters that are proportionality constants related to the capacitive and diffusion-limited processes, respectively. Figure 6f shows the *C*_m_ and fraction of current attributed to two different charge-storage processes. The capacitive effects are significantly higher than the diffusion-controlled contributions from the electrode, indicating that the large *C*_m_ of NPC-800 electrode mainly originates from the capacitive response. The capacitive response of the NPC-800 electrode is almost constant over a wide range of scan rates (10–200 mV s^−1^), further demonstrating the excellent rate performance of NPC-800. As shown in Figs. S8 and S9, when the pyrolysis temperature is insufficient (less than 800 °C), the diffusion-controlled capacitance ratio is relatively high. That is, the NPCs obtained at a low temperature with a lower specific surface area and conductivity hinder the transport of ions and electrons. As the temperature increases (≥ 800 °C), the specific surface area increases and the conductivity is enhanced. Hence, diffusion is no longer the factor limiting the capacitance. NPC-800 has a smaller specific surface area and lower electrical conductivity than NPC-1000 and NPC-1100, but a higher C_m_, and could contribute to better surface wettability due to the higher N content (Table S2) [23, 55–57].

## Conclusions

In summary, we prepared N-containing polymer precursors via mechanochemical polymerization without a solvent or catalyst, followed by pyrolysis and the removal of the SiO_2_ template to obtain hierarchical micro-mesoporous NPCs. Owing to the sufficient hierarchical pore structure, high specific surface area, and nitrogen doping, the NPC-1000 prepared by this method exhibited excellent ORR electrocatalytic activity, stability, and methanol tolerance. When used as a ZAB cathode, NPC-1000 exhibited excellent discharge performance comparable to that of Pt/C. Furthermore, its discharge stability is much better than that of Pt/C. The NPC-800 prepared by the same method also exhibited excellent supercapacitance performance due to its high specific capacity (256 F g^−1^ at 0.5 A g^−1^ and 431 F g^−1^ at 10 mV s^−1^). A high rate performance and excellent cycling stability (98.7% retention after 10,000 cycles at 10 A g^−1^) in an aqueous 6-M KOH solution were observed. This study confirmed the feasibility of preparing nitrogen-containing polymers by ball milling to prepare NPCs with high electrocatalytic activity, which could replace noble-metal electrocatalytic materials.

## Electronic supplementary material

Below is the link to the electronic supplementary material.

Supplementary material 1 (DOCX 757 kb)
