# Supplementary material for: Hierarchical N-Doped Porous Carbons for Zn–Air Batteries and Supercapacitors
Source: Nanomicro Lett. 2020 Jan 10;12:20. doi: 10.1007/s40820-019-0364-z (PMC7770743; doi:10.1007/s40820-019-0364-z)
Supplement: Supplementary file 1 — Supplementary material 1 (DOCX 757 kb) [file 40820_2019_364_MOESM1_ESM.docx]

**Supporting Information:**

**Hierarchical N-Doped Porous Carbons for Zn–Air Batteries and Supercapacitors**

Beibei Guo,^1,3^ Ruguang Ma,^1^ Zichuang Li,^1^ Shaokui Guo,^1^ Jun Luo,^3^ Minghui Yang,^2,4^ Qian Liu,^1,2^ Tiju Thomas,^5^ and Jiacheng Wang ^1,2 *^

^1^ State Key Laboratory of High Performance Ceramics and Superfine Microstructure, Shanghai Institute of Ceramics, Chinese Academy of Sciences, Shanghai 200050, China

^2^ Center of Materials Science and Optoelectronics Engineering, University of Chinese Academy of Sciences, Beijing 100049, China

^3^ School of Materials Science and Engineering, Shanghai University, Shanghai 200444, China

^4^ Solid State functional Materials Research Laboratory, Ningbo Institute of Materials Technology and Engineering, Chinese Academy of Sciences, Ningbo 315201, China

^5^ Department of Metallurgical and Materials Engineering, Indian Institute of Technology Madras, Adyar, Chennai 600036, Tamil Nadu, India

^*^**Email:** [jiacheng.wang@mail.sic.ac.cn](mailto:jiacheng.wang@mail.sic.ac.cn)

*Preparation of Ni-Fe layered double hydroxide (NiFe-LDH)*

The Ni-Fe layered double hydroxide (NiFe-LDH) was fabricated by a hydrothermal method reported previously [S1]. Typically, 3.6 mmol Ni (NO_3_)_2_ • 6H_2_O, 1.8 mmol Fe(NO_3_)_3_ • 9H_2_O and 126 mmol CO(NH_2_)_2_ were dissolved in 50 mL of deionized wate and stirred for 15 min. subsequently, the mixtures were hydrothermal treatment at 120 °C for 10 h. The resulting products were washed with deionized water and ethanol 3 times, and dried at 60 °C for 12 h.


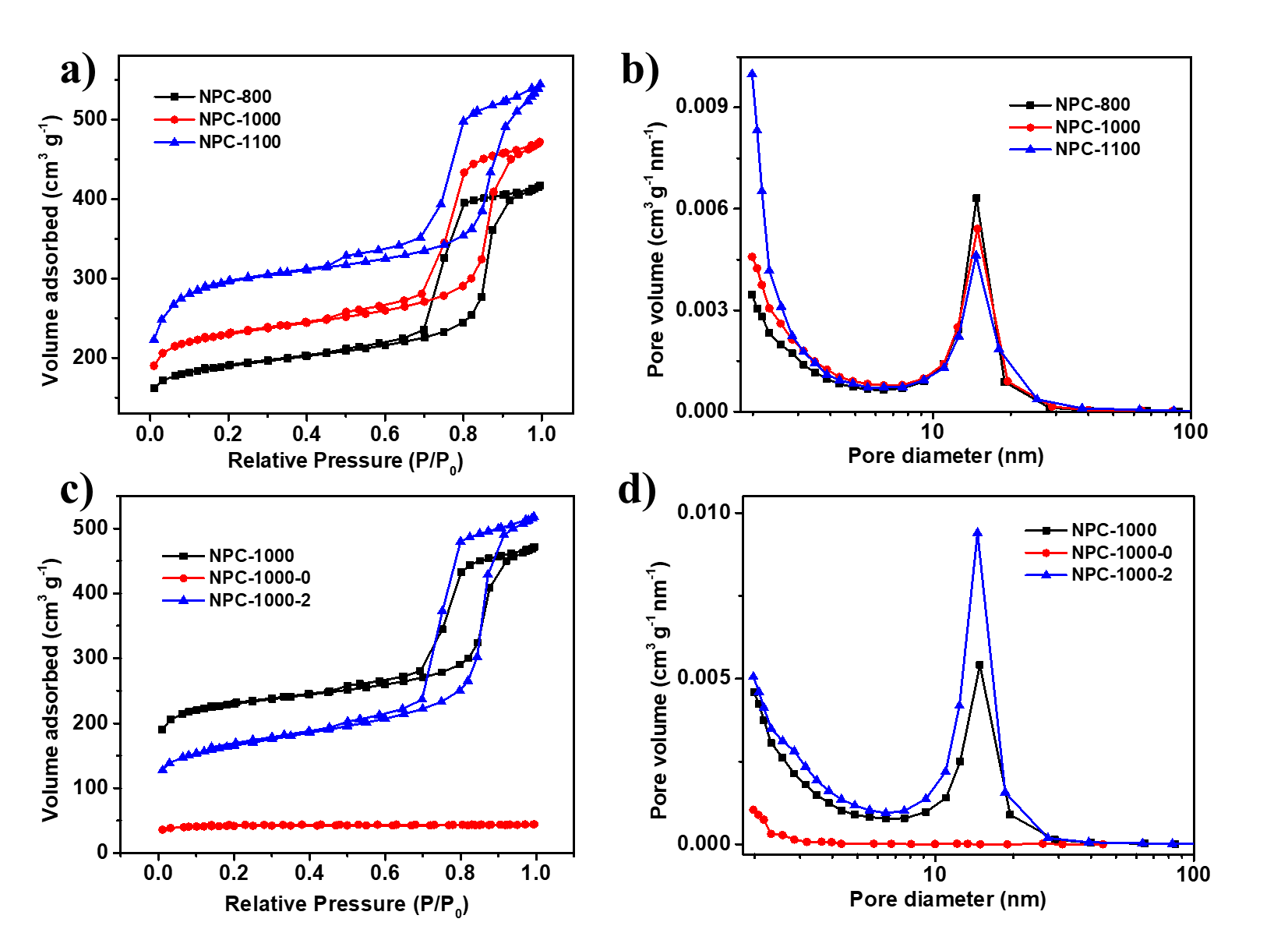


**Fig. S1** a) Nitrogen adsorption-desorption isotherms and b) pore-size distribution of NPC-800, NPC-1000 and NPC-1100, indicating that surface area (S_BET_) and micropore area were gradually improved with the increasing of temperature; c) Nitrogen adsorption-desorption isotherms and b) pore-size distribution of NPC-1000, NPC-1000-0 and NPC-1000-2, implying that the introduction of SiO_2_ template greatly increased the specific surface area of NPC, but excessive SiO_2_ would lead to collapse of pore structure.


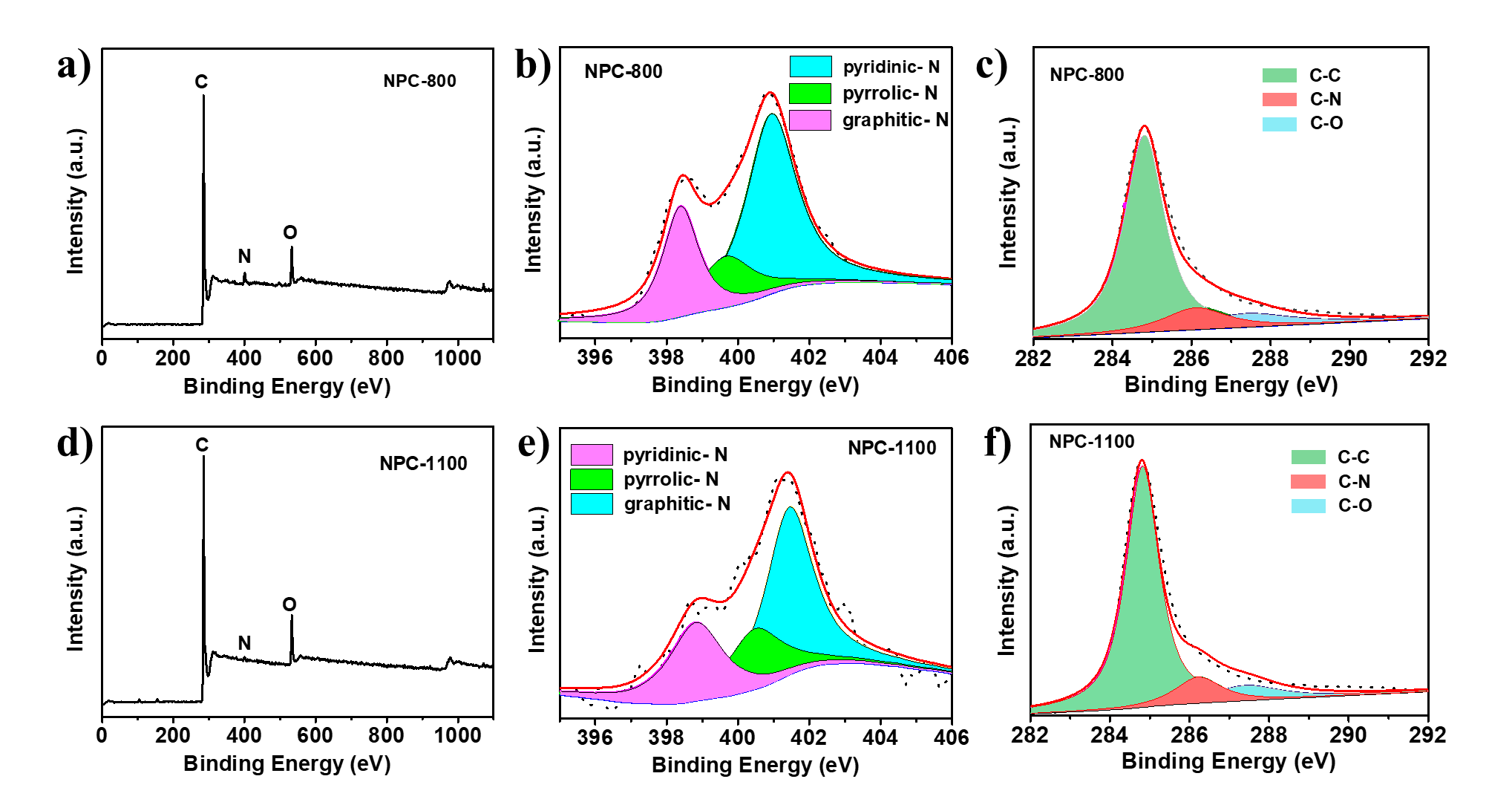


**Fig. S2** a) The XPS full spectrum, b) high-resolution N 1s, and c) C1s of NPC-800; d) The XPS full spectrum, e) high-resolution N 1s, and f) C1s of NPC-1100.


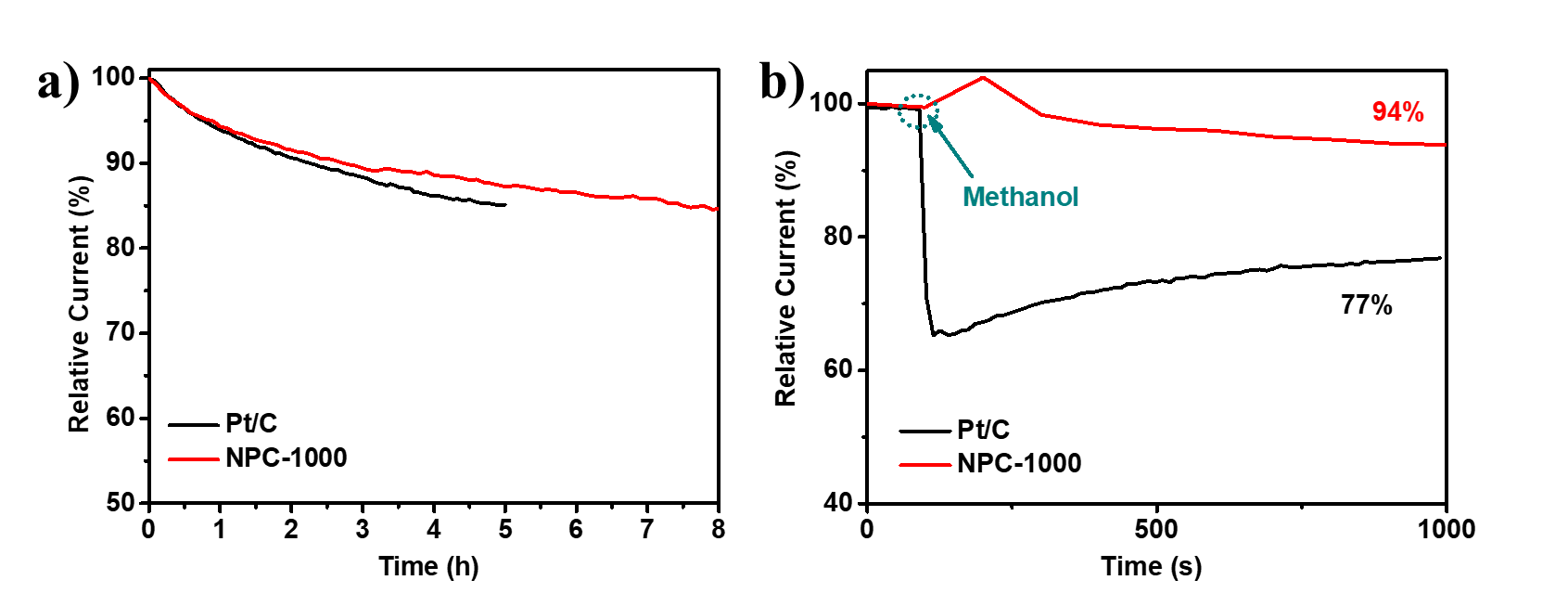


**Fig. S3** a) Current–time (i-t) chronoamperometric responses for NPC-1000 and 20% Pt/C at 0.4 V (vs. RHE) for the ORR at a rotating speed of 1600 rpm; b) comparison of chronoamperometric responses for NPC-1000 and commercial Pt/C in O_2_-saturated 0.1 M KOH electrolyte with 3 M methanol added at 100 s.

**Fig. S4** ORR polarization curves in O_2_-saturated 0.1 M KOH (rotation rate: 1600 rpm).


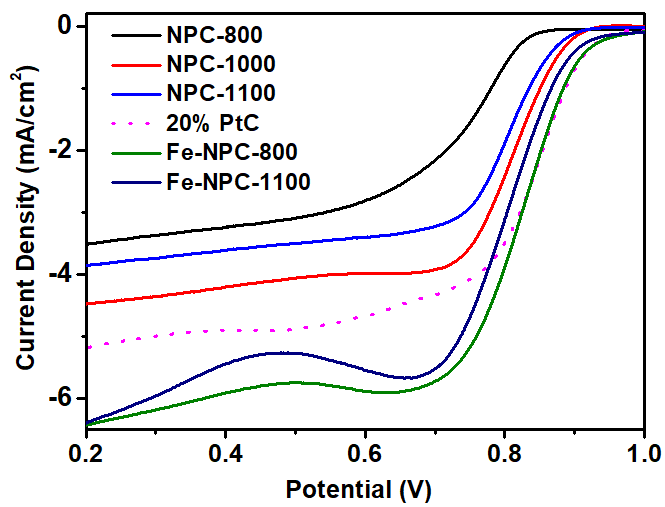


**Fig. S5** ORR polarization curves in O_2_-saturated 0.1 M KOH (rotation rate: 1600 rpm) indicating the porous carbon prepared by ball-milling and pyrolysis is also a good metal carrier.


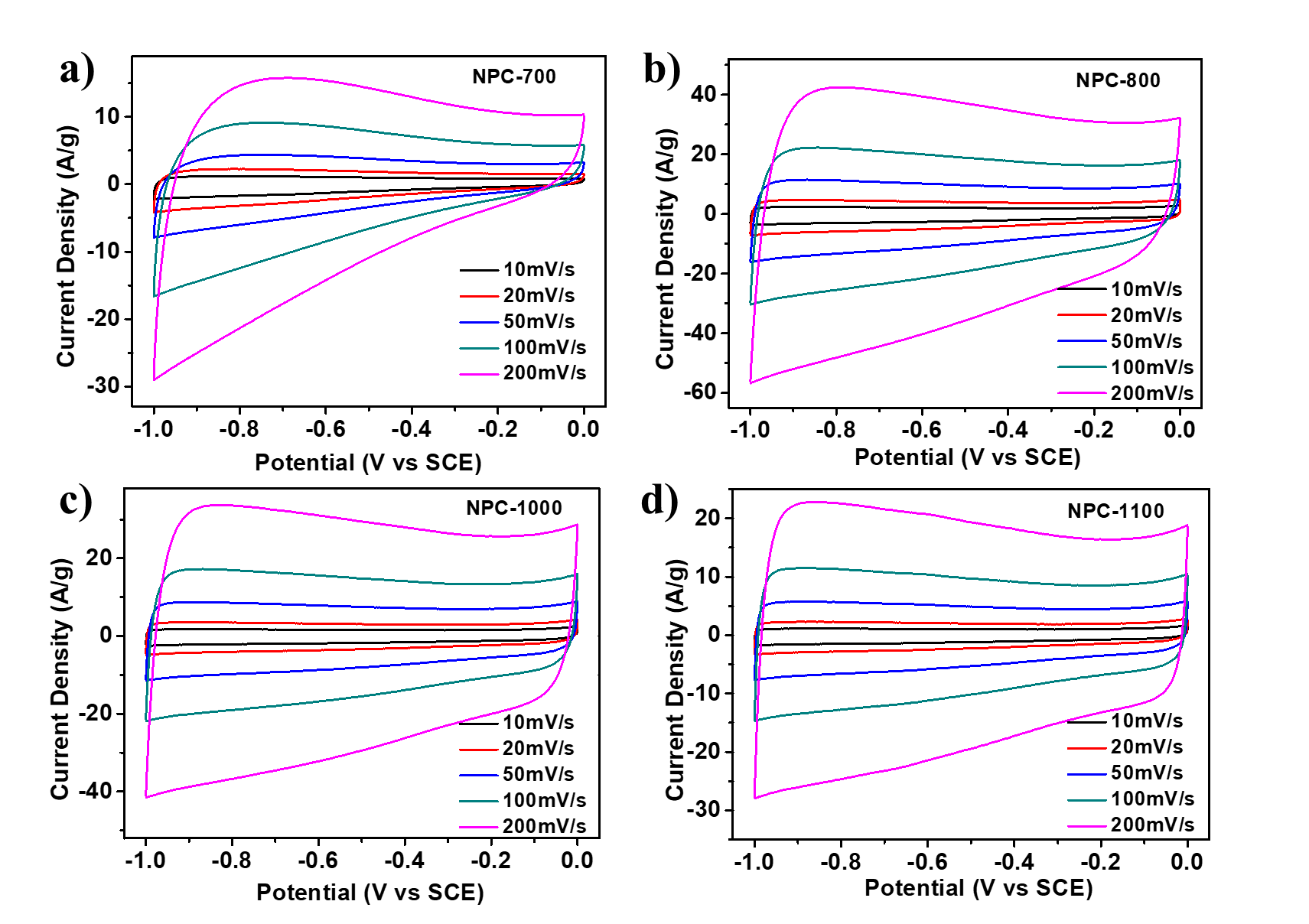


**Fig. S6** CV curves of a) NPC-700, b) NPC-800, c) NPC-1000 and d) NPC-1100 at different scan rates in 6M KOH solution.

**
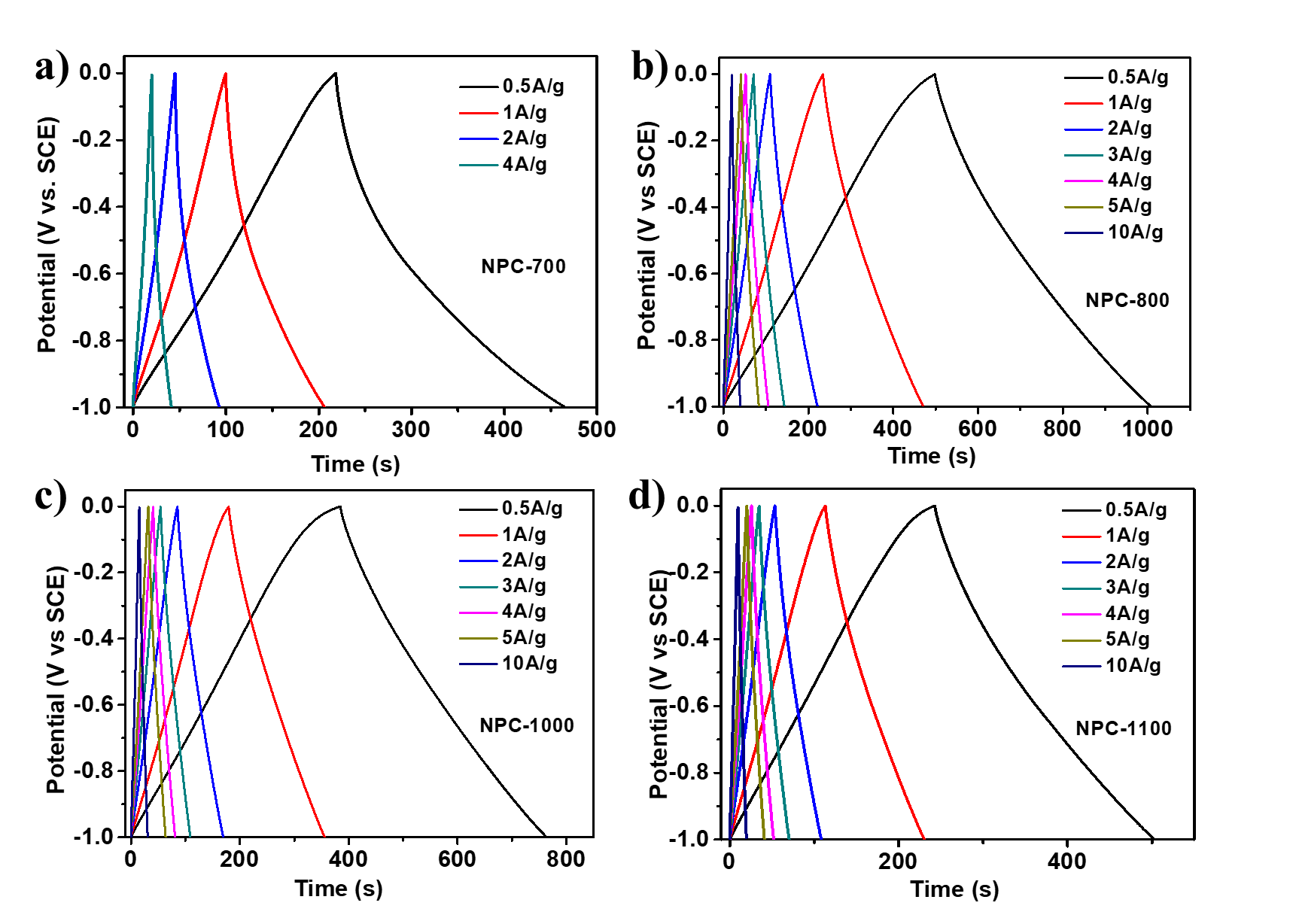
**

**Fig. S7** Galvanostatic charge-discharge (GCD) curves of a) NPC-700, b) NPC-800, c) NPC-1000 and d) NPC-1100 at different current density in 6M KOH solution.

**
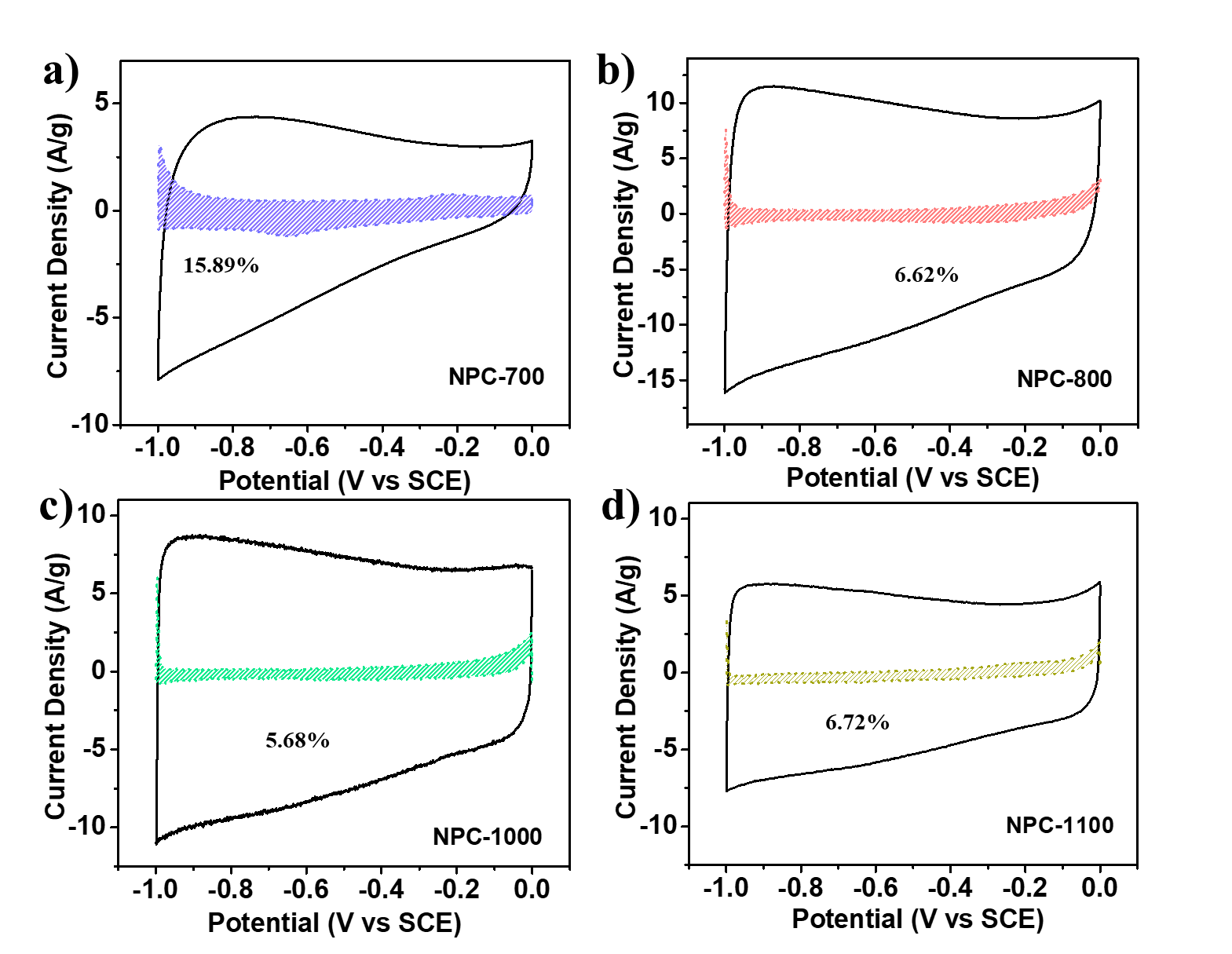
**

**Fig. S8** Deconvolution of diffusion-controlled (shaded area) and capacitive-like capacitance in a) NPC-700, b) NPC-800, c) NPC-1000 and d) NPC-1100 at 50 mV/s.

**
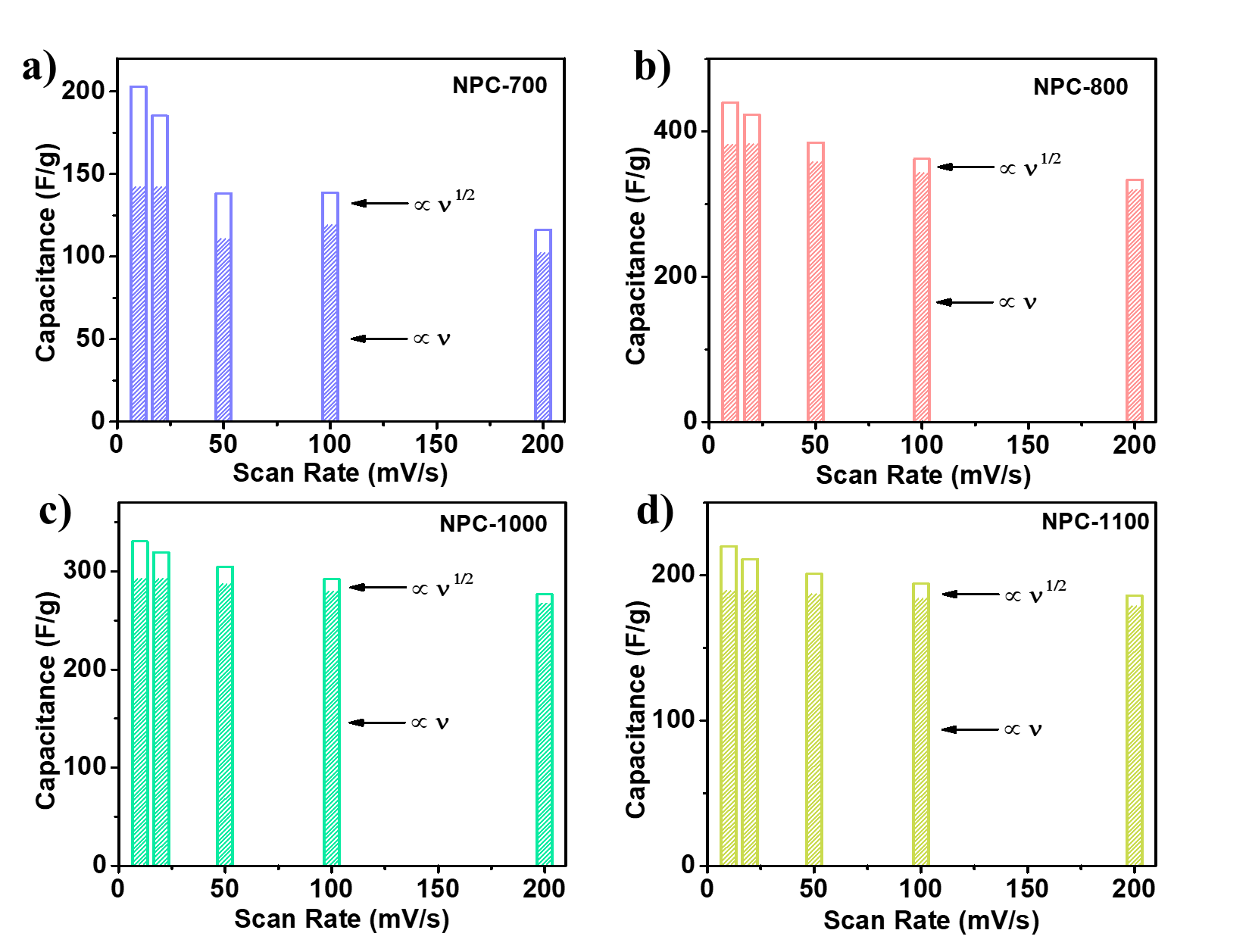
**

**Fig. S9** Deconvolution of charge contribution as a function of scan rates.

**Table S1.** The surface area, pore volume and pore size of the as prepared catalysts.

| **Samples** | **BET Surface Area**  **(m^2^/g)** | **Micropore Area(m^2^/g)** | **Pore Volume**  **(cm^3^/g)** | **Micropore Volume**  **(cm^3^/g)** | **Pore Size**  **(nm)** |
| --- | --- | --- | --- | --- | --- |
| NPC-800 | 641.3 | 464.0 | 0.64 | 0.22 | 3.97 |
| NPC-1000 | 777.8 | 564.7 | 0.72 | 0.26 | 3.70 |
| NPC-1100 | 1013.2 | 684.0 | 0.82 | 0.31 | 3.23 |
| NPC-1000-0 | 139.6 | 118.1 | 0.07 | 0.05 | 1.95 |
| NPC-1000-2 | 569.1 | 303.0 | 0.79 | 0.14 | 5.55 |

**Table S2.** A comparison in contents of Pyridinic N, Pyrrolic N and Quaternary N species for resultant NPC-800 and NPC-1000 and NPC-1100 catalysts calculated from their N 1s spectra.

| **Samples** | **Pyridinic N**  **(at %)** | **Pyrrolic N**  **(at %)** | **Quaternarty N**  **(at %)** | **Total N Content**  **(at %)** |
| --- | --- | --- | --- | --- |
| NPC-800 | 0.24 | 0.16 | 0.60 | 4.33 |
| NPC-1000 | 0.20 | 0.16 | 0.64 | 2.18 |
| NPC-1100 | 0.27 | 0.15 | 0.58 | 1.08 |

**Table S3.** Comparison of the ORR electrocatalyst performances of the prepared catalysts in 0.1 M KOH.

| **Catalysts** | | **E_onset_ (V)** | **E_1/2_ (V)** |
| --- | --- | --- | --- |
| NPC-700 | 0.83 | | 0.69 |
| NPC-800 | 0.84 | | 0.73 |
| NPC-900 | 0.86 | | 0.75 |
| NPC-1000 | 0.9 | | 0.82 |
| NPC-1100 | 0.88 | | 0.80 |
| PtC | 0.93 | | 0.85 |

**Table S4.** Summary of the ORR performances of reported heteroatom-doped porous carbons in 0.1 M KOH.

| **Catalysts** | **E_onset_ (V vs RHE)** | **E_1/2_ (V vs RHE)** | **References** |
| --- | --- | --- | --- |
| NPC-1000 | 0.9 | 0.82 | **This work** |
| N-doped C/CNTs-1000 | 0.92 | 0.82 | Angew. Chem. Int. Ed., 2014, 53, 4102-4106 |
| NPMC-1000 | 0.94 | 0.85 | Nat. Nanotechnol., 2015, 10, 444-452. |
| CNF@NC | unknown | 0.72 | Appl. Surf. Sci.,2017, 443,266–273 |
| a-C NH3 900 | 0.83 (900rpm) | ~0.7 (900rpm) | Small, 2019, 1902081 |
| Ultra N-doped carbon nanosheet | 0.95 | 0.82 | Energy Environ. Sci.2019, 12,332 |

**Reference for SI**

[S1] Zhong H, Liu T, Zhang S, Li D, Tang P, Alonso-Vante N, Feng Y. Template-free synthesis of three-dimensional nife-ldh hollow microsphere with enhanced oer performance in alkaline media. J. Energy Chem. **33**(130-137 (2019). doi:10.1016/j.jechem.2018.09.005
